# Temperature-dependent changes in the host-seeking behaviors of parasitic nematodes

**DOI:** 10.1186/s12915-016-0259-0

**Published:** 2016-05-06

**Authors:** Joon Ha Lee, Adler R. Dillman, Elissa A. Hallem

**Affiliations:** Department of Microbiology, Immunology, and Molecular Genetics, University of California, Los Angeles, California 90095 USA; Department of Nematology, University of California, Riverside, California 92521 USA

**Keywords:** Parasitic nematodes, Entomopathogenic nematodes, Host-seeking behavior, Olfaction, Olfactory plasticity

## Abstract

**Background:**

Entomopathogenic nematodes (EPNs) are lethal parasites of insects that are of interest as biocontrol agents for insect pests and disease vectors. Although EPNs have been successfully commercialized for pest control, their efficacy in the field is often inconsistent for reasons that remain elusive. EPN infective juveniles (IJs) actively search for hosts to infect using a diverse array of host-emitted odorants. Here we investigate whether their host-seeking behavior is subject to context-dependent modulation.

**Results:**

We find that EPN IJs exhibit extreme plasticity of olfactory behavior as a function of cultivation temperature. Many odorants that are attractive for IJs grown at lower temperatures are repulsive for IJs grown at higher temperatures and vice versa. Temperature-induced changes in olfactory preferences occur gradually over the course of days to weeks and are reversible. Similar changes in olfactory behavior occur in some EPNs as a function of IJ age. EPNs also show temperature-dependent changes in their host-seeking strategy: IJs cultured at lower temperatures appear to more actively cruise for hosts than IJs cultured at higher temperatures. Furthermore, we find that the skin-penetrating rat parasite *Strongyloides ratti* also shows temperature-dependent changes in olfactory behavior, demonstrating that such changes occur in mammalian-parasitic nematodes.

**Conclusions:**

IJs are developmentally arrested and long-lived, often surviving in the environment through multiple seasonal temperature changes. Temperature-dependent modulation of behavior may enable IJs to optimize host seeking in response to changing environmental conditions, and may play a previously unrecognized role in shaping the interactions of both beneficial and harmful parasitic nematodes with their hosts.

**Electronic supplementary material:**

The online version of this article (doi:10.1186/s12915-016-0259-0) contains supplementary material, which is available to authorized users.

## Background

The widespread use of chemical insecticides in agriculture is a growing health and ecological concern, with an increasing number of studies revealing detrimental effects on organisms such as non-pest insects, aquatic animals, and humans [1–4]. Commonly explored alternatives to synthetic chemical pesticides include natural pesticides and transgenic plants expressing insecticidal protectants [5, 6]. However, natural pesticides often present a toxicity concern and genetically modified organisms remain a topic of public debate and controversy [4–8].

Entomopathogenic nematodes (EPNs) of the genera *Steinernema* and *Heterorhabditis* are lethal parasites of insects that are used throughout the world as biocontrol agents for insect pests [9]. EPNs offer a number of advantages as biocontrol agents – they are found on all continents except Antarctica and are therefore considered non-invasive [10], kill a wide variety of insect agricultural pests [11], are amenable to low-cost mass production [12], and are considered safe to humans and the environment [13]. However, while EPNs are used successfully against some insect pests [14], their efficacy in the field is often inconsistent, precluding their widespread use [14–16]. A number of variables have been found to influence their effectiveness, including temperature, humidity, soil type, and timing of application [16]. Nevertheless, reliable predictors of field efficacy remain lacking.

**Fig. 1:**
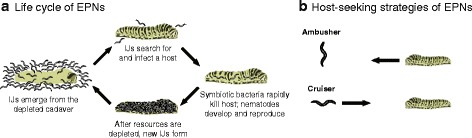
Life cycle and host-seeking strategies of entomopathogenic nematodes (EPNs). **a** The EPN life cycle. Infective juveniles (IJs) search for hosts in the environment. Once a host is identified, IJs infect the host and release endosymbiotic bacteria harbored in their gut, which rapidly kill the host [48]. IJs then resume development and reproduce until resources are depleted, at which point new IJs form and emerge from the host cadaver to search for new hosts. Adapted from Hallem et al*.* [22]. **b** Host-seeking strategies of EPNs. Ambushers wait for hosts to approach; cruisers migrate through their environment in search of hosts [21]

Host-seeking behavior is a critical aspect of EPN biology that is poorly understood in relation to biocontrol. Like many parasitic nematodes, EPNs infect insects as infective juveniles (IJs), which are developmentally arrested third-stage larvae analogous to the dauer larvae of free-living nematodes [17] (Fig. 1a). IJs are long-lived, non-feeding, and stress-resistant [17–19], and seek out hosts using chemosensory cues [20, 21]. They are attracted to the respiratory byproduct carbon dioxide (CO_2_) as well as a diverse array of host-specific odorants, with different species showing different odor response profiles [22–24]. IJs are also attracted to plant odorants, including some that are emitted in response to insect predation [25–29]. A comparison of olfactory behavior in EPNs and other parasitic nematodes revealed that olfactory preferences reflect host specificity rather than phylogeny [22, 30], suggesting that olfaction plays an important role in host selection for these parasites.

EPNs vary not only in their olfactory preferences, but also in their host-seeking strategies. Some are cruisers that actively search for hosts, some are ambushers that attack passing hosts, and some use an intermediate strategy (Fig. 1b) [31, 32]. Despite differences in host-seeking strategy, all EPNs show robust chemotaxis to live hosts and host-derived odorants [22–24, 30, 33–35]. Thus, although ambushers and cruisers show different behaviors in the absence of host stimuli, both are capable of migrating toward hosts in response to host-emitted sensory cues.

During the course of previous experiments examining the responses of EPNs to host-emitted odorants, we were surprised to discover that olfactory behavior appeared to vary depending on IJ cultivation temperature and age. Temperature and age are known to modulate feeding, mating, learning and memory, and other biological processes in many different animals [36–44]. However, little is known about whether they alter olfactory sensory valence – i.e.*,* whether an odorant is attractive or repulsive – or foraging strategy. To investigate this further, we conducted an in-depth analysis of the effects of cultivation temperature and IJ age on the host-seeking behaviors of six EPN species.

We found that EPNs showed dramatic changes in olfactory behavior and host-seeking strategy as a function of cultivation temperature and age. Temperature-induced changes were reversible over the course of days to weeks. We also found that one of the EPNs tested, the cricket specialist *Steinernema scapterisci,* showed age-dependent changes in CO_2_ response that correlated with changes in the response to insect hosts. Thus, both generalist and specialist EPNs exhibit extreme olfactory plasticity as a function of temperature and/or age. To test whether olfactory plasticity occurs in other types of parasitic nematodes, we examined the skin-penetrating rat parasite *Strongyloides ratti*, a close relative of the human threadworm *Strongyloides stercoralis*. We found that *Str. ratti* also exhibits temperature-dependent olfactory plasticity, suggesting that olfactory plasticity may be widespread among parasitic nematodes.

Taken together, our results demonstrate that host seeking is context-dependent and describe a novel behavioral plasticity with broad implications for the interactions of both beneficial and harmful parasitic nematodes with their hosts. Temperature and age often vary during commercial application of EPNs, and our results suggest that this variability will cause changes in host-seeking behavior that may alter the efficacy of EPNs as biocontrol agents. In addition, parasitic nematodes of humans, livestock, and plants cause extensive disease and economic loss worldwide [45–47]. Our results raise the possibility that a better understanding of their olfactory plasticity could enable the development of targeted odor-based traps or repellents for preventing harmful infections.

## Results

### *Steinernema carpocapsae* olfactory responses are modulated by temperature

We first examined the olfactory behavior of *Steinernema carpocapsae*, an EPN with a broad geographical distribution that is widely used for insect control [14]. To test whether cultivation temperature affects the olfactory behavior of *Ste. carpocapsae* IJs, we cultured nematodes at either 15 °C, 20 °C, or 25 °C and examined the olfactory responses of IJs 4 weeks after emergence from the insect host. Responses were tested in a chemotaxis assay, in which IJs were allowed to migrate toward or away from an odorant source (Additional file 1: Figure S1). We examined the responses of IJs to a chemically diverse panel of odorants that included known EPN attractants and repellents as well as odorants previously found to be emitted by potential insect hosts [22, 23].

**Fig. 2:**
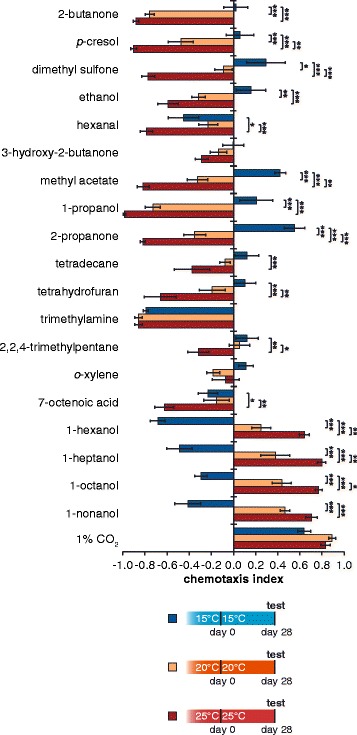
*Steinernema carpocapsae* shows temperature-dependent differences in olfactory behavior. Infective juveniles were cultured at 15 °C, 20 °C, or 25 °C and tested 4 weeks after host-emergence. Dramatic differences in olfactory behavior were observed as a function of cultivation temperature. * *P* < 0.05; ** *P* < 0.01; *** *P* < 0.001; two-way ANOVA with Tukey’s post-test; n = 6–16 trials for each condition. Error bars represent standard error of the mean (SEM). Mean, n, and SEM values for each condition are listed in Additional file 7: Dataset S1

We found that IJs showed dramatic differences in their olfactory preferences depending on their cultivation temperature. The responses to 80 % of the tested odorants changed depending on the cultivation temperature of the IJs, and nearly half of these changes resulted in responses of opposite valence (Fig. 2). For example, methyl acetate and 2-propanone elicited attractive responses from 15 °C IJs, repulsive responses from 25 °C IJs, and intermediate responses from 20 °C IJs. The reverse was true for long-chain alcohols such as 1-hexanol and 1-heptanol, which were repulsive for 15 °C IJs but attractive for 20 °C and 25 °C IJs. Changes in olfactory behavior occurred gradually across temperature ranges, such that IJs exhibited intermediate responses for intermediate temperature points (Additional file 2: Figure S2A). Furthermore, these changes in response valence with IJ cultivation temperature were seen regardless of the assay temperature; for example, IJs cultivated at 15 °C showed similar olfactory preferences when assayed at both 15 °C and room temperature (Additional file 2: Figure S2B). Thus, the observed differences in response valence among cultivation conditions were not due to acute acclimation to the assay temperature.

### *Ste. carpocapsae* exhibits temperature-induced olfactory plasticity

**Fig. 3:**
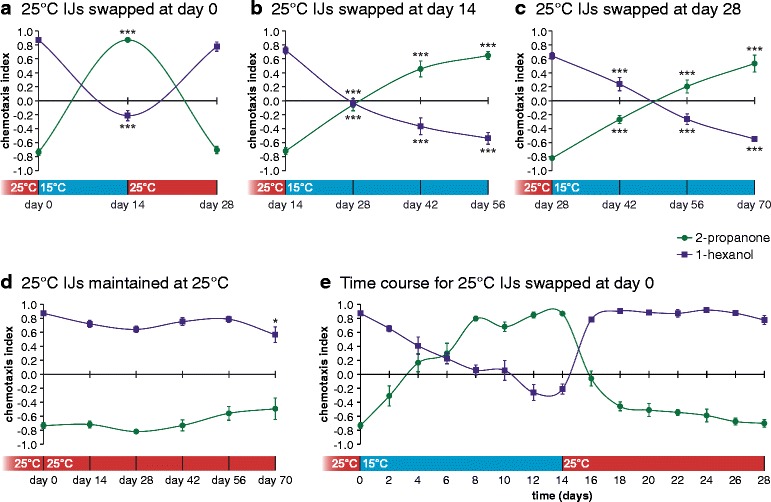
*Steinernema carpocapsae* exhibits temperature-induced olfactory plasticity. **a**–**c** Temperature-swapping infective juveniles (IJs) from 25 °C to 15 °C, or vice versa, reversed their olfactory preferences. Olfactory plasticity occurred more rapidly in younger IJs (**a**) than older IJs (**b**, **c**). *** *P* < 0.001 relative to day 0 IJs; two-way ANOVA with Dunnett’s post-test; *n* = 6–22 trials for each condition. **d** The olfactory responses of IJs maintained at 25 °C remained relatively unchanged over a 70-day period, although the response to 1-hexanol at day 70 was slightly reduced. Day 28 data are taken from Fig. 2. * *P* < 0.05 relative to day 0 IJs; two-way ANOVA with Dunnett’s post-test; *n* = 6–22 trials for each condition. **e** Temperature-induced olfactory plasticity occurred gradually over the course of days; *n* = 6–22 trials for each time point. For all graphs, error bars represent standard error of the mean (SEM). Mean, *n*, and SEM values for each assay are listed in Additional file 7: Dataset S1

We next tested whether temperature changes can alter olfactory behavior. For this experiment, nematodes were cultured at 25 °C and day 0 IJs were tested for their responses to 2-propanone and 1-hexanol, two odorants that showed dramatic temperature-dependent valence changes in opposite directions. As expected, 2-propanone was strongly repulsive and 1-hexanol was strongly attractive (Fig. 3a). IJs were then temperature-swapped to 15 °C, maintained at 15 °C for 2 weeks, and tested on day 14. After 2 weeks at 15 °C, IJs showed dramatically different olfactory responses: 2-propanone was strongly attractive and 1-hexanol was neutral. Day 14 IJs were then temperature-swapped back to 25 °C and tested at 4 weeks, at which point olfactory responses had shifted back: 2-propanone was again strongly repulsive and 1-hexanol was strongly attractive (Fig. 3a). Thus, temperature changes induce reversible changes in olfactory behavior. Similar plasticity was observed with older IJs, although olfactory responses changed more slowly in older IJs than younger IJs (Fig. 3b, c). By contrast, 2-propanone remained repulsive and 1-hexanol remained attractive for IJs maintained at 25 °C (Fig. 3d).

The robust differences in response valence between IJs maintained at 25 °C and IJs temperature-swapped from 25 °C to 15 °C were observed regardless of the assay temperature (Additional file 2: Figure S2C). Dose-response analysis for the different IJ populations revealed that olfactory preferences were consistent across CO_2_ and odorant concentrations (Additional file 3: Figure S3A, B), indicating that temperature changes primarily alter valence. IJs exhibited olfactory plasticity in response to blends of odorants at lower concentrations (Additional file 3: Figure S3C), suggesting the results for individual odorants can be generalized to odorant mixtures. In addition to affecting response valence, temperature and age may also affect response sensitivity, as 25 °C IJs aged for 14 days showed increased attraction to 0.5 % CO_2_ and increased repulsion to low concentrations of 2-propanone (Additional file 3: Figure S3A, B). To investigate effects on sensory valence rather than sensitivity, we chose to focus on 1 % CO_2_ and undiluted odorants for all further experiments. Changes in olfactory preferences occurred in individual IJs and in multiple strains of *Ste. carpocapsae*, and were not affected by IJ cultivation density (Additional file 4: Figure S4). Taken together, these results demonstrate that individual IJs exhibit olfactory plasticity in response to temperature changes.

We then examined the rate at which olfactory responses change in temperature-swapped IJs by testing responses to 2-propanone and 1-hexanol every 2 days following the temperature swap. We found that temperature-induced changes in olfactory preferences occurred gradually over the course of days to weeks (Fig. 3e). These results are consistent with olfactory plasticity being a response to seasonal, but not diurnal, temperature variation.

### Olfactory responses are stable in the absence of a temperature change

To examine the stability of IJ olfactory responses in the absence of a temperature change, we examined the behavior of IJs that were cultured at room temperature, temperature-swapped to 15 °C on day 0, and maintained at 15 °C for up to 4 months. We found that, after the initial change in olfactory behavior induced by the temperature swap, olfactory preferences remained unchanged for up to 4 months (Additional file 5: Figure S5). Thus, olfactory preferences exhibit long-term stability under constant temperature conditions.

### Temperature- and age-dependent changes in olfactory behavior occur broadly across EPN species

EPNs of the genera *Steinernema* and *Heterorhabditis* are distantly related but have similar lifestyles as a result of convergent evolution [48, 49]. Despite their similar lifestyles, different EPNs show different odor response profiles, which are thought to reflect their host specificities [22, 23]. To test whether olfactory plasticity occurs in EPNs other than *Ste. carpocapsae*, we examined the responses of five additional species – *Steinernema riobrave*, *Steinernema feltiae*, *Steinernema scapterisci*, *Heterorhabditis bacteriophora*, and *Heterorhabditis indica* ─ under the same cultivation conditions to an 8-odorant panel comprised of odorants that were previously shown to elicit robust responses from different EPNs [22, 30]. *Ste. feltiae*, *H. bacteriophora*, and *H. indica* have broad geographical distributions and are used to control a wide variety of insect pests [50], while *Ste. riobrave* is a warm-adapted strain with a narrower geographical distribution and host range [50]. *Ste. scapterisci* is a specialist known to preferentially infect crickets and has been shown to be effective in lowering populations of *Scapteriscus* spp. mole crickets in Florida [51, 52]. For these experiments, nematodes were cultured at 25 °C and IJs were either maintained at 25 °C or temperature-swapped to 15 °C after emergence from the host. For comparison, we examined the responses of *Ste. carpocapsae* to the same eight-odorant panel under these conditions.

**Fig. 4:**
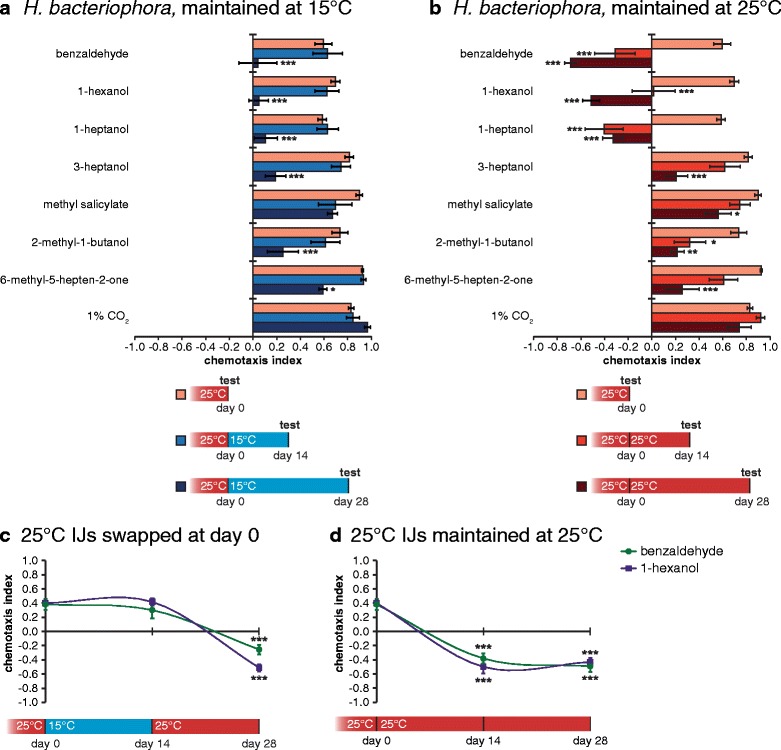
*Heterorhabditis bacteriophora* exhibits age-dependent changes in olfactory behavior. **a** Infective juveniles (IJs) were grown at 25 °C and temperature-swapped to 15 °C on day 0. The olfactory responses of temperature-swapped day 28 IJs but not day 14 IJs were significantly different from those of 25 °C day 0 IJs for all odorants except methyl salicylate and CO_2_. * *P* < 0.05; *** *P* < 0.001 relative to day 0 IJs; two-way ANOVA with Dunnett’s post-test; *n* = 6–8 trials for each condition. **b** IJs were grown and maintained at 25 °C. Olfactory responses to all odorants except CO_2_ changed as a function of parasite age. * *P* < 0.05; ** *P* < 0.01; *** *P* < 0.001 relative to day 0 IJs; two-way ANOVA with Dunnett’s post-test; *n* = 6–8 trials for each condition. **c**, **d** IJs were grown at 25 °C and either temperature-swapped to 15 °C and back to 25 °C (**c**) or maintained at 25 °C (**d**). IJs were then tested for their responses to benzaldehyde and 1-hexanol, which showed the largest valence change in (**b**). In both conditions, IJs switched their odorant response from attraction to repulsion over time, consistent with an age-dependent effect. *** *P* < 0.001 relative to day 0 IJs; two-way ANOVA with Dunnett’s post-test; *n* = 8 trials for each condition. For all graphs, error bars represent standard error of the mean (SEM). Mean, *n*, and SEM values for each assay are listed in Additional file 7: Dataset S1

Similar to what we observed with our larger odorant panel, we found that the olfactory responses of *Ste. carpocapsae* IJs cultured at 25 °C and then temperature-swapped to 15 °C changed dramatically, while the olfactory responses of IJs maintained at 25 °C remained relatively unchanged. Half of the tested odorants were attractive to 25 °C IJs regardless of age, but repulsive or neutral to IJs temperature-swapped from 25 °C to 15 °C (Additional file 6: Figure S6A). By contrast, *H. bacteriophora* olfactory responses changed primarily as a function of age rather than temperature – responses were not significantly different following the temperature change but were significantly different in older IJs regardless of the cultivation temperature (Additional file 6: Figure S6B and Fig. 4a, b). For example, all of the tested odorants except CO_2_ elicited significantly different responses from 25 °C IJs at day 0 versus day 28, and three of the eight odorants elicited responses of opposite valence (Fig. 4b). Age-dependent changes were less extreme at 15 °C within the time frame examined (Fig. 4a), most likely because age-related changes occur more slowly at 15 °C than 25 °C. Temperature-swap experiments with 25 °C IJs swapped to 15 °C and back to 25 °C showed a switch in valence over time (Fig. 4c). This change in olfactory behavior was also observed with IJs maintained at 25 °C (Fig. 4d). These results are consistent with *H. bacteriophora* showing age-dependent changes in olfactory preferences.

The four other EPNs tested – *Ste. scapterisci*, *Ste. riobrave*, *H. indica*, and *Ste. feltiae* – exhibited both age- and temperature-dependent changes in olfactory behavior, although to varying extents (Additional file 6: Figure S6C–F). For example, temperature effects were seen with the response of *Ste. scapterisci* to 1-heptanol, *Ste. riobrave* to methyl salicylate, *H. indica* to 1-hexanol, and *Ste. feltiae* to CO_2_, where IJs temperature-swapped to 15 °C showed significant differences from 25 °C IJs. On the other hand, age effects were seen with the response of *Ste. scapterisci* to methyl salicylate, *Ste. riobrave* to benzaldehyde, *H. indica* to 6-methyl-5-hepten-2-one, and *Ste. feltiae* to 2-methyl-1-butanol, where day 0 and 14 IJs showed significant differences regardless of temperature treatment. Taken together, these data suggest that temperature- and age-dependent effects in olfactory behavior are broadly conserved across EPN species, though the extent of these changes varies across different species and different odorants. Moreover, these results demonstrate that IJs show age-dependent changes in sensory behavior even though they are developmentally arrested.

### *Ste. scapterisci* shows an age-dependent change in its response to CO_2_ and insect hosts

CO_2_ is emitted by all aerobic organisms, including insects, and is strongly attractive for EPNs as well as many other parasitic animals [21–23]. Moreover, CO_2_ is an essential host cue for EPNs; in the absence of CO_2_, attraction to host odor blends is greatly reduced or eliminated [22–24, 53]. Most of the species tested were robustly attracted to CO_2_ regardless of temperature or age, consistent with CO_2_ being an essential host cue (Figs. 2, 4, Additional file 3: Figure S3A and Additional file 6: Figure S6). Because it is so ubiquitous in nature, it is thought of as a general host cue. Thus, the stable attraction to CO_2_ may direct parasitic worms to live animals, whereas olfactory plasticity may allow for context-dependent modulation of host discrimination. However, we were surprised to find that the cricket specialist *Ste. scapterisci* was repelled by CO_2_ under the conditions tested (Additional file 6: Figure S6C), which was in contrast to our previous finding that *Ste. scapterisci* is attracted to CO_2_ [23]. We therefore wondered whether the CO_2_ response of *Ste. scapterisci* might vary with cultivation temperature or parasite age.

**Fig. 5:**
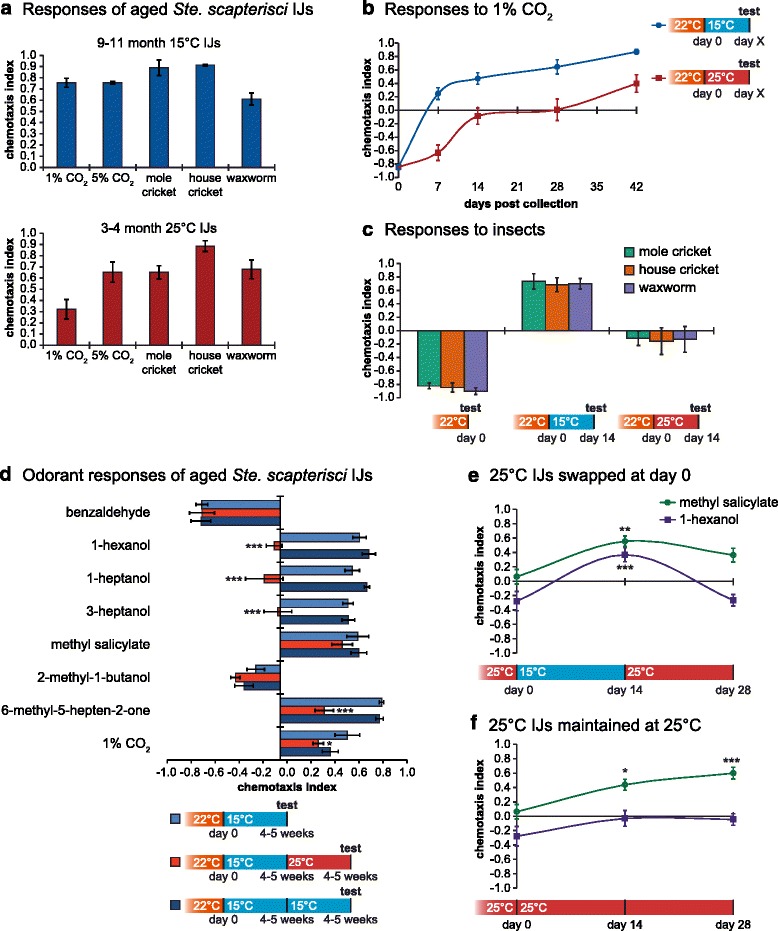
*Steinernema scapterisci* shows temperature- and age-dependent changes in response to odorants, CO_2_, and host odor. **a** Infective juveniles (IJs) incubated at 15 °C or 25 °C for several months showed attraction to CO_2_ and insects. *n* = 4–8 trials for each condition. **b** IJs were grown at room temperature (22 °C ± 1 °C) and incubated at either 15 °C or 25 °C. For both temperature treatments, IJs showed a gradual shift in their CO_2_ response from repulsive to attractive with increasing age; *n* = 8–16 trials for each condition. **c** IJs showed a shift in their responses to potential insect hosts that corresponded with their changing response to CO_2_; *n* = 6–8 trials for each condition. **d** IJs were grown at room temperature (22 °C ± 1 °C) and incubated at 15 °C for 4–5 weeks until their responses to CO_2_ shifted to attraction. IJs were then swapped to 25 °C or maintained at 15 °C for another 4–5 weeks. The responses of IJs to several of the tested odorants differed as a function of temperature, suggesting olfactory plasticity occurs independently of the change in CO_2_ response valence. * *P* < 0.05; *** *P* < 0.001 relative to the initial 4–5 week IJ population; two-way ANOVA with Dunnett’s post-test; *n* = 6 trials for each condition. **e**, **f** IJs were grown at 25 °C and either temperature-swapped to 15 °C and back to 25 °C (**e**) or maintained at 25 °C (**f**) and tested for their responses to two selected odorants. Responses to these odorants changed as a function of age and/or temperature. * *P* < 0.05; ** *P* < 0.01; *** *P* < 0.001 relative to day 0 IJs; two-way ANOVA with Dunnett’s post-test; *n* = 8 trials for each condition. For all graphs, error bars represent standard error of the mean (SEM). Mean, *n*, and SEM values for each assay are listed in Additional file 7: Dataset S1

We first looked at *Ste. scapterisci* IJs that were aged for several months at either 15 °C or 25 °C. We found that, in both cases, old IJs showed strong attraction to CO_2_ as well as potential insect hosts, including its natural host, the mole cricket *Scapteriscus borellii* (Fig. 5a). These results corroborated our previous findings and indicated that *Ste. scapterisci* changes the valence of its CO_2_ response over time. To investigate how quickly this change occurs, we took room temperature-grown *Ste. scapterisci* IJs, cultivated them at 15 °C or 25 °C, and measured their response to 1 % CO_2_ over the course of 6 weeks (Fig. 5b). We found that, while there was a temperature-dependent difference in the rate at which the responses changed, both IJs maintained at 15 °C and those maintained at 25 °C gradually shifted their CO_2_ response from repulsion to attraction. Furthermore, this change in CO_2_ response correlated with a change in their response to insect hosts (Fig. 5c). Taken together, these data suggest that *Ste. scapterisci* changes its response to both CO_2_ and insect hosts in an age-dependent manner.

We next asked whether temperature-induced changes in olfactory behavior occurred independently of the age-dependent changes in CO_2_ response. To address this, we cultivated IJs at 15 °C for 4–5 weeks until they became attracted to CO_2_ and then measured their responses to our selected eight-odorant panel (Fig. 5d). We then temperature-swapped these IJs to 25 °C or maintained them at 15 °C for another 4–5 weeks. The aged worms, which were consistently attracted to CO_2_, showed significant temperature-dependent changes in olfactory behavior. We also tested 25 °C IJs that were either swapped to 15 °C and then back to 25 °C, or maintained at 25 °C, in response to two odorants that were selected based on their mode of valence change (Fig. 5e, f). Methyl salicylate showed a predominantly age-dependent change, whereas 1-hexanol showed a temperature-dependent change. Taken together, these data suggest that *Ste. scapterisci* IJs exhibit dramatic changes in olfactory behavior in response to both temperature and age.

### Cultivation temperature alters parasite host-seeking strategy

**Fig. 6:**
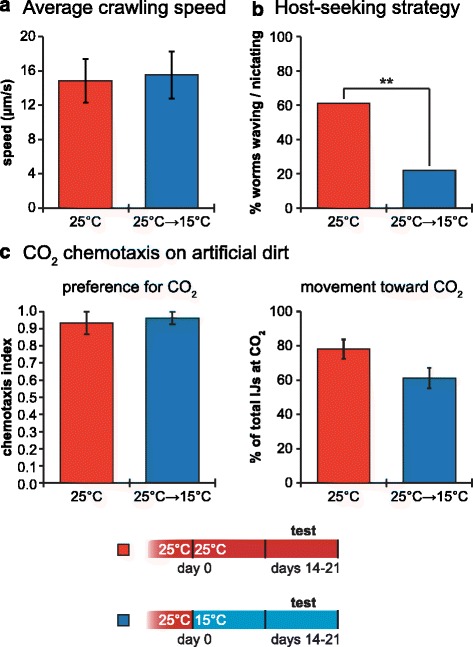
*Steinernema carpocapsae* exhibits temperature-induced changes in host-seeking strategy. **a** Temperature changes did not affect the crawling speeds of infective juveniles (IJs). The crawling speeds of day 14–21 IJs that were either maintained at 25 °C, or temperature-swapped from 25 °C to 15 °C on day 0, were not significantly different (Mann–Whitney test); *n* = 26–31 IJs for each condition. **b** Temperature changes altered the host-seeking strategies of IJs. Day 14–21 IJs that were maintained at 25 °C showed a greater propensity to nictate than IJs that were temperature-swapped from 25 °C to 15 °C on day 0. ** *P* < 0.01; Fisher’s exact test; *n* = 36 IJs for each condition. **c** Temperature changes did not alter CO_2_ chemotaxis behavior. CO_2_ attraction for IJs that were either maintained at 25 °C, or temperature-swapped from 25 °C to 15 °C on day 0, was not significantly different (left graph, unpaired *t*-test). In addition, the percentage of the population that migrated toward the CO_2_ was not significantly different for the two treatment conditions (right graph, unpaired *t*-test); *n* = 6–8 trials for each condition. For all graphs, error bars represent standard error of the mean (SEM). Mean, *n*, and SEM values for each assay are listed in Additional file 7: Dataset S1

In addition to odor-driven behavior, another critical aspect of host finding is host-seeking strategy. We therefore asked whether host-seeking strategy is also modulated by cultivation temperature using *Ste. carpocapsae*, a species classically categorized as an ambusher [32]. We first examined the motility of 2- to 3-week-old IJs that were either cultured at 25 °C, or cultured at 25 °C and temperature-swapped to 15 °C immediately after emergence from the host. Using automated worm tracking [30], we found that both sets of IJs showed similar average crawling speeds (Fig. 6a). We then examined nictation, an ambushing behavior in which the IJ stands on its tail and waves its head to facilitate host attachment [32]. Nictation was assayed on “micro-dirt” chips containing near-microscopic raised agar pillars as an artificial dirt substrate [54], since IJs cannot nictate on standard agar plates due to the high surface tension on the plate. We found that IJs maintained at 25 °C had a greater propensity to nictate than IJs temperature-swapped from 25 °C to 15 °C (Fig. 6b). These results suggest that host-seeking strategy is temperature-dependent, with *Ste. carpocapsae* behaving more like an ambusher at higher temperatures and more like a cruiser at lower temperatures.

The fact that *Ste. carpocapsae* IJs showed a greater tendency to ambush at warmer temperatures raised the question of whether they remained attracted to host-emitted odorants under these conditions. Previous studies demonstrated chemotaxis of *Ste. carpocapsae* IJs to host-emitted odorants at room temperature on flat agar surfaces [22, 23]; however, IJs were not capable of nictating under these assay conditions. To test whether *Ste. carpocapsae* IJs chemotax toward host-emitted odorants at warm temperatures when given a choice between nictating and crawling, we examined IJ behavior in a CO_2_ gradient on micro-dirt chips. We found that both IJs maintained at 25 °C and IJs temperature-swapped from 25 °C to 15 °C displayed equally robust chemotaxis toward CO_2_ (Fig. 6c). Thus, although warm-temperature IJs nictated more than cold-temperature IJs in the absence of a host stimulus, both warm- and cold-temperature IJs migrated toward a host stimulus to similar extents. These results suggest that *Ste. carpocapsae* IJs display cruising behavior at lower temperatures, and a combination of cruising and ambushing behavior at higher temperatures. A flexible switch in foraging behavior, combined with changes in olfactory behavior, may allow these IJs to optimize host-seeking under changing environmental conditions.

### Temperature-dependent olfactory plasticity also occurs in mammalian-parasitic nematodes

**Fig. 7:**
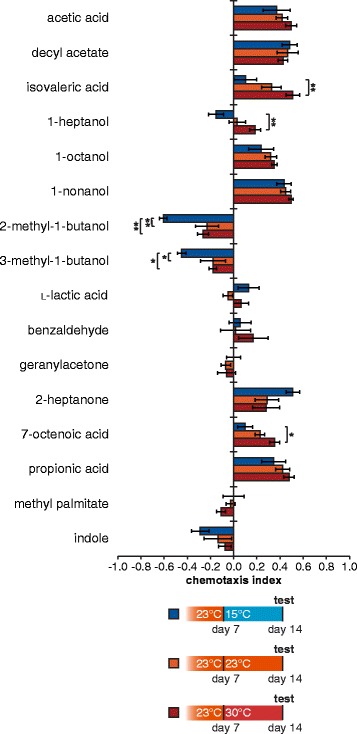
*Strongyloides ratti*, a skin-penetrating mammalian parasite, shows temperature-dependent changes in olfactory behavior. Nematodes were allowed to develop to the infective juvenile (IJ) stage at 23 °C, and IJs were then incubated for 7 days at either 15 °C, 23 °C, or 30 °C. IJs incubated at 15 °C showed significantly different responses to some odorants from IJs incubated at 23 °C or 30 °C. * *P <* 0.05; ** *P* < 0.01; two-way ANOVA with Tukey’s post-test; *n* = 6–10 trials for each condition. Error bars represent standard error of the mean (SEM). Mean, *n*, and SEM values for each assay are listed in Additional file 7: Dataset S1

We next asked if temperature-dependent changes in olfactory behavior occur in other types of parasitic nematodes. To address this question, we turned to another parasitic nematode that engages in active host seeking, the skin-penetrating rat parasite *Strongyloides ratti. Str. ratti* is a close relative of the human threadworm *Strongyloides stercoralis*, a parasite estimated to infect up to 100 million humans worldwide [55]. We selected a 16-odorant panel of mammalian-derived odorants, many of which were previously identified as attractants for *Str. ratti* or *Str. stercoralis* [30, 56]*,* and examined the effect of cultivation temperature on *Str. ratti* olfactory behavior. We found that IJs temperature-swapped from 23 °C to 15 °C showed significant differences in their responses to several odorants compared to IJs maintained at 23 °C or temperature-swapped from 23 °C to 30 °C (Fig. 7). In particular, isovaleric acid and 7-octenoic acid elicited attractive responses for IJs cultivated at 30 °C but neutral responses for those cultivated at 15 °C. Thus, like EPNs, skin-penetrating nematodes exhibit temperature-dependent olfactory plasticity. *Str. ratti* has been reported to show seasonal prevalence in wild rats [57], raising the possibility that temperature-dependent changes in olfactory behavior could contribute to seasonal cycles of infectivity.

## Discussion

Our results demonstrate that parasitic nematodes modulate their olfactory preferences and host-seeking strategy in response to changing external cues such as temperature and internal cues such as age. Environmental temperature and age have been shown to modulate olfactory sensitivity or discrimination in a number of free-living animals. For example, Oriental fruit moths show a decreased ability to discriminate among pheromone blends at higher temperatures [58], and aged *Caenorhabditis elegans* adults show decreased attraction to benzaldehyde [59]. Temperature also modulates olfactory discrimination in turtles [60, 61], olfactory sensitivity in the fruit fly *Drosophila melanogaster* [62, 63]*,* and CO_2_ sensitivity in *C. elegans* adults [64]. However, the robust, long-lasting temperature-induced olfactory valence changes that occur in parasitic nematodes have not been previously reported.

The finding that temperature-dependent changes in IJ olfactory behavior occur over a period of several days to weeks suggests that these changes are reflective of a seasonal adaptation. EPNs inhabit a broad range of geographic habitats, many of which are subject to seasonal temperature variation [10], and persist in the soil throughout the year [65–67]. In changing seasonal conditions, temperature-dependent olfactory plasticity may allow EPNs to modulate or optimize their host seeking by altering host preferences, enabling EPNs to target seasonally prevalent hosts or host life stages. Most EPNs are capable of parasitizing a wide variety of insect pests [11, 68], many of which show seasonal variation in their prevalence or the prevalence of particular life stages [69–71]. For example, the Western corn rootworm *Diabrotica virgifera virgifera* and the bluegrass billbug *Sphenophorus parvulus* are known hosts for *Ste. carpocapsae* [14, 72] and both have seasonal life cycles [73, 74]. Olfactory plasticity may also allow EPNs to migrate toward seasonal plants that insect hosts infest, since several of the odorants that were subject to temperature-dependent changes, including benzaldehyde, 1-hexanol, 1-hexanal, and methyl salicylate, are emitted by plants as well as insects [75]. Furthermore, volatiles emitted from plants vary depending on temperature or season [76, 77]. Thus, temperature-induced changes in host-seeking strategy could allow EPNs to compensate for temperature-induced changes in insect movement or habitat, or to target both mobile and sedentary hosts or host life stages.

In contrast to the other parasite species tested, *Ste. scapterisci* and *Str. ratti* have very specific host ranges [51, 78, 79]. Olfactory plasticity in these parasites may instead reflect an adaptation to changing host volatile emissions. Both mammals and insects show variations in their volatile emissions as a result of seasonal changes in photoperiod or hormonal levels [80–84]. Thus, temperature-dependent olfactory plasticity may allow parasites with specific host ranges to locate and infect preferred hosts under changing environmental conditions. We note that fewer temperature-dependent changes in olfactory behavior were observed with *Str. ratti* than with some of the EPN species (Fig. 7 and Additional file 6: Figure S6). In future studies, it will be interesting to test whether other mammalian-parasitic species show more extreme temperature-dependent changes in olfactory behavior, or whether extreme temperature-dependent olfactory plasticity is limited to some EPNs.

*Ste. scapterisci* differed from the other EPNs tested in that its response to CO_2_ changed with IJ age – young IJs were repelled by CO_2_, while older IJs were attracted to it (Fig. 5). The changes in CO_2_ response paralleled changes in the response to host odor, including the odor of its natural host, the mole cricket (Fig. 5). Thus, the response to CO_2_ may be driving the response to hosts. The repulsion of young IJs from CO_2_ and host odor may function as a dispersal mechanism, causing the young IJs to migrate out into the environment rather than remain in the vicinity of already utilized host niches. Long-range dispersal mechanisms may be particularly important for *Ste. scapterisci* due to the faster speed of crickets relative to the insect larvae targeted by most EPNs. Another possibility is that young *Ste. scapterisci* IJs may be risk-averse, as has been proposed for other *Steinernema* species [85, 86]. Mole crickets are refractory to infection, even by *Ste. scapterisci* [23], and it may be that only as the IJs age and lose energy resources are they likely to attempt infecting a mole cricket.

While we have found that long-lasting changes in olfactory behavior occur in several parasitic nematode IJs, it remains unknown whether the analogous dauer stage of free-living nematodes exhibits olfactory plasticity. Previous studies investigating *C. elegans* and the beetle-associated necromenic nematode *Pristionchus pacificus* and age- or temperature-dependent changes in olfactory behavior have looked at adults but not dauers [42, 59, 87, 88]. Like parasitic nematode IJs, *C. elegans* dauers exhibit robust chemotaxis toward odorants [22, 30]; the chemotaxis behaviors of the dauers of other free-living species have not yet been examined in depth. *P. pacificus* live in close association with beetles and engage in active host-seeking [87], while C*. elegans* dauers show phoretic associations that appear to be relatively non-specific [89]. In future studies, it will be interesting to determine whether these species also show context-dependent changes in host-seeking behaviors.

Although EPNs are generally categorized as either ambushers or cruisers [11, 90], a number of recent studies suggest that the traditional ambusher/cruiser distinction may be an oversimplification. For example, the classical ambusher *Ste. carpocapsae* is effective at controlling a number of immobile insect pests, including large pine weevil larvae and codling moth larvae, suggesting it can employ a cruising strategy to target immobile hosts [91]. *Ste. carpocapsae* also disperses more in some types of soil than others, suggesting that host-seeking strategy is substrate-dependent [91]. Our results demonstrate that host-seeking strategy is also regulated by temperature, with warmer temperatures stimulating ambushing and colder temperatures stimulating cruising (Fig. 6b). However, even warm-temperature IJs crawl toward the source of an odorant (Fig. 6c), demonstrating that the decision to ambush or cruise depends on both temperature and the presence of host-emitted sensory cues. Our results argue against the simple ambusher/cruiser distinction and suggest that host-seeking strategy is context-dependent.

The cellular mechanisms that underlie olfactory plasticity in parasitic nematodes remain obscure, as remarkably little is known about the mechanisms of olfaction in parasitic nematodes. The only olfactory neurons that have so far been identified in parasitic nematodes are the CO_2_-sensing BAG neurons [22]. However, since nematode neuroanatomy and function are often conserved across species [21], the positional analogs of the *C. elegans* olfactory neurons are likely to function as olfactory neurons in parasitic nematodes. In *C. elegans*, the AWC olfactory neurons are also thermosensitive [92, 93]. Thus, if olfactory neuron function is similar in parasitic nematodes, the integration of olfactory and thermosensory cues may occur at the level of the primary sensory neurons. The molecular mechanisms that mediate olfactory plasticity in parasitic nematodes also remain to be elucidated. However, we note that temperature-dependent changes in development or metabolism are unlikely to account for olfactory plasticity since the observed changes in olfactory behavior are reversible, occur gradually over the course of days to weeks following a temperature swap, and occur more slowly in older IJs (Fig. 3).

In addition to responding to olfactory and thermosensory stimuli, parasitic nematodes respond to other sensory modalities such as electric fields [94, 95], magnetic fields [96], and vibration [97, 98]. These sensory modalities have been proposed to play a role in host seeking [94–98], and in future studies it will be interesting to test whether they are subject to context-dependent plasticity. In addition, in the case of EPNs, IJ populations exhibit behavioral heterogeneity. For example, dispersal behavior varies among individuals and depends on the time of emergence from the insect host [99]. Host-invasion behavior also varies among individuals such that some IJs invade new hosts more readily than others [85]. Thus, physiological cues other than age also regulate host seeking. The nature of these cues and how they are integrated with temperature, age, and olfactory environment remain to be investigated.

## Conclusions

Our results uncover a surprising behavioral plasticity in EPNs that is likely to influence their efficacy for insect biocontrol. Future studies of the relationship between temperature, parasite age, and field efficacy may improve the utility of EPNs for biocontrol. In addition, our finding that skin-penetrating nematodes exhibit similar behavioral plasticity raises the possibility that context-dependent behavioral changes are widespread among parasitic nematodes. Parasitic nematodes of humans cause some of the most common neglected tropical diseases, particularly in low resource areas, while parasitic nematodes of livestock and plants are a major cause of economic and food loss worldwide [45, 100]. A better understanding of behavioral plasticity in these parasites could facilitate the development of new strategies for preventing harmful infections.

## Methods

### Nematodes

EPN strains were as follows: *Ste. carpocapsae* All [22, 23] (except for DD136 and Sal [101] in Additional file 4: Figure S4C); *H. bacteriophora* M31e [22, 23]; *Ste. scapterisci* FL [23]; *Ste. riobrave* TX [23]; *Ste. feltiae* SN [102, 103]; and *H. indica* Hom1 [103]. All strains were from our laboratories except for *Ste. carpocapsae* DD136, *Ste. carpocapsae* Sal, and *H. indica* Hom1, which were generously provided by David Shapiro-Ilan, USDA.

### Nematode culturing

For all EPNs except *Ste. scapterisci,* 3–6 last-instar *Galleria mellonella* larvae (purchased commercially) were placed in a 5-cm Petri dish lined with a 55-mm Whatman 1 filter paper; 250 μL of ddH_2_O containing 1000–2000 IJs was distributed evenly on the filter paper. The Petri dishes were placed at 15 °C, 20 °C, room temperature (22 °C ± 1 °C), or 25 °C for 9–11 days (20 °C, room temperature, or 25 °C) or 20–22 days (15 °C) and then placed on White traps [104]. IJs were collected after 3–4 days (20 °C, room temperature, or 25 °C) or 4–6 days (15 °C) and rinsed three times in ddH_2_O. The day of collection was considered day 0. IJs were stored at 15 °C, 20 °C, room temperature, or 25 °C suspended in 10 mL ddH_2_O in 50-mL tissue culture flasks unless otherwise indicated.

For *Ste. scapterisci*, adult *Acheta domesticus* house crickets (purchased commercially) were placed individually in 5-cm Petri dishes lined with a 55-mm Whatman 1 filter paper; 250 μL of ddH_2_O containing ~250–1000 IJs was distributed evenly on the filter paper. The Petri dishes were placed at room temperature or in 25 °C incubators for 4–13 days and then placed on White traps. IJs were collected after 3–11 days and rinsed three times in ddH_2_O. The day of the collection was considered day 0. Populations were collected within 4 days of IJ formation and emergence. IJs were stored at 15 °C or 25 °C suspended in 10 mL ddH_2_O in 50-mL tissue culture flasks.

*Str. ratti* was cultured essentially as previously described [30]. Briefly, rats were subcutaneously inoculated with ~800 IJs in 300 μL sterile PBS. Fecal pellets containing nematode eggs were collected from patent rats. Fecal-charcoal plates were prepared by mixing the fecal pellets with dH_2_O and autoclaved charcoal (bone char, Ebonex) in an approximately 1:1 ratio of feces to charcoal. The mix was poured into Petri dishes (100 mm by 20 mm) lined with wet 90 mm Whatman 1 filter paper and stored at 23 °C until use. IJs were isolated from fecal-charcoal plates using a Baermann apparatus [105] after 7 days. IJs were then rinsed three times in dH_2_O and stored in ~7.5 mL BU saline [105] in 50-mL tissue culture flasks at 15 °C, 23 °C, or 30 °C for 7 days before use. Protocols and procedures for the host passage of *Str. ratti* in rats were approved by the UCLA Office of Animal Research Oversight (Protocol No. 2011-060-12C), which adheres to the AAALAC standards for laboratory animal use, and were in strict accordance with the Guide for the Care and Use of Laboratory Animals.

For temperature-swap experiments, nematodes were cultured at room temperature or 25 °C as indicated. IJs were then split into the different temperature populations (15 °C or 25 °C for EPNs; 15 °C, 23 °C, or 30 °C for *Str. ratti*).

### Chemotaxis assays

Odorant, host, and CO_2_ chemotaxis assays were performed as previously described [22, 23, 30]. Assays were performed on chemotaxis assay plates [106]. The assay setup is shown in Additional file 1: Figure S1. For odorant chemotaxis assays, 1 μL (for EPNs) or 2 μL (for *Str. ratti*) of 5 % sodium azide was placed in the center of each scoring region as an anesthetic; 5 μL of odorant (Sigma-Aldrich or Fisher) was placed in the center of one scoring region and 5 μL of a control (paraffin oil or ddH_2_O) was placed in the center of the other scoring region. Liquid odorants were tested undiluted unless otherwise noted. Solid odorants were dissolved as follows: *p-*cresol and methyl palmitate, 0.05 g in 2.5 mL paraffin oil; dimethyl sulfone and 3-hydroxy-2-butanone, 1 g in 4 mL ddH_2_O; l-lactic acid, 0.05 g in 2.5 mL ddH_2_O; indole, 0.05 g in 2.5 mL absolute ethanol. Trimethylamine was purchased as a ~45 wt. % in H_2_O (Sigma-Aldrich). For the dose-response curves in Additional file 3: Figure S3B, 1-hexanol was diluted in paraffin oil and 2-propanone in ddH_2_O. The odor blends in Additional file 3: Figure S3C were created as follows: Mix 1: 10^–1^ dilutions of methyl acetate, 1-propanol, and 2-propanone in ddH_2_O; Mix 2: 10^–1^ dilutions of 1-hexanol, 6-methyl-5-hepten-2-one, and methyl salicylate in paraffin oil; Mix 3: 10^–1^ dilutions of 1-hexanol, 7-octenoic acid, and 2,2,4-trimethylpentane in paraffin oil. For CO_2_ chemotaxis assays, 50 mL gastight syringes were filled with a certified mixture containing 1 % CO_2_ or 0 % CO_2_ (control syringe) in a 10 % oxygen (O_2_) or 21 % O_2_, balance nitrogen background. For host chemotaxis assays, one adult mole cricket (*Scapteriscus borellii*, collected from the wild as previously described [23, 107]), four adult house crickets, or six waxworms were placed in a 50-mL gastight syringe and tested against a control syringe filled with room air. Syringes were depressed using a syringe pump initially at a rate of 5 mL/min for at least 1 minute to flush the tubing, then at a rate of 0.5 mL/min for the duration of the assay. Gases were delivered to opposite sides of the assay plate through holes drilled into the plate lids directly above the centers of the scoring regions.

For each trial, 2–3 μL of worm pellet containing ~100–500 nematodes was washed three times with ddH_2_O and placed in the center of the assay plate. Assay plates were left undisturbed on a vibration-reducing platform and were scored after 1 hour for CO_2_ and host assays or 3 hours for odor assays. A chemotaxis index (CI) was calculated as CI = (number of worms in odorant or CO_2_ circle – number of worms in control circle)/(number of worms in both circles) (Additional file 1: Figure S1). Trials were always done in pairs, and were only counted if more than seven worms moved into the scoring region for both trials and if the CI difference between the two trials was less than one to account for directional bias due to room vibration. For all chemotaxis assays, at least two replicates of trial pairs were performed using separate batches of worms on different days to account for batch-to-batch and day-to-day variability. All chemotaxis assays except those in Additional file 2: Figure S2B, C were conducted at room temperature; thus, we examined the effects of cultivation temperature rather than current ambient temperature on olfactory preferences.

For the assay temperature controls in Additional file 2: Figure S2B, C, sodium azide and paraffin oil were applied to chemotaxis plates as above and kept at the assay temperature (15 °C, room temperature, or 25 °C) for at least 1 hour; 15 °C or 25 °C IJs were washed three times in 15 °C or 25 °C ddH_2_O, respectively, then placed on the pre-incubated chemotaxis plates along with 15 °C or 25 °C odorant. Throughout the set-up, pre-incubated reagent and IJ exposure to room temperature was minimized and kept to under 5 minutes. Assays were then conducted at room temperature as described above or in 15 °C or 25 °C incubators.

For the experiment shown in Additional file 4: Figure S4A, where the same IJs were tested under multiple conditions, 25 °C day 0 *Ste. carpocapsae* IJs were tested for their response to 2-propanone by using a modified chemotaxis “lid” assay in which the odorant and control were placed on a small piece of Whatman 1 filter paper affixed above the scoring region [30]. No sodium azide was used. After 3 hours, IJs initially repelled by 2-propanone were removed from the one-third plate region at the control side of the plate. Recovered IJs were stored at either 15 °C or 25 °C in 96-well plates in 50 μL of ddH_2_O for 2 weeks before re-testing their response to 2-propanone in a chemotaxis assay where the scoring regions were modified as illustrated to calculate a response index (Additional file 4: Figure S4A).

### Automated worm tracking

Automated worm tracking was performed essentially as previously described [30]. Briefly, 5–10 IJs from three replicate batches were placed on a chemotaxis plate and allowed to acclimate to room temperature for 1 hour. To measure average speed, IJ movement was recorded using an Olympus E-PM1 digital camera attached to a Leica S6 D microscope for 60 s. Recordings were analyzed using WormTracker and WormAnalyzer software (Miriam Goodman lab, Stanford University) [108]. Analysis was conducted as previously described [30], except that the following WormTracker settings were adjusted: sample rate = 15 frames/s; auto-thresholding correction factor = 0.20. The average speed of IJs that did not move during the assay period but were confirmed to be alive at the end of the assay was manually zeroed. We note that one *Ste. carpocapsae* IJ in the 25 °C to 15 °C temperature-swapped population crawled at a speed that was approximately 10 times faster than that of the other IJs tested, suggesting it was a “sprinter” [109]. Sprinters, which comprise less than 5 % of the IJ population, disperse farther than other IJs and are thought to facilitate long-range population dispersal in the absence of a host [109]. This IJ was not included in our population analysis.

### Nictation assay

Assays were conducted on ~3 × 3.5 cm “micro-dirt” agar chips cast from polydimethylsiloxane (PDMS) molds as previously described [54]. Micro-dirt chips were made by pouring 4 % agar dissolved in ddH_2_O onto a PDMS mold containing arrays of near-microscopic posts (25 μm post height, 25 μm post radius, 25 μm distance between posts). Chips were dried at 37 °C for 2 hours before use. IJs were allowed to acclimate to room temperature for ~30 minutes before ~5 IJs from 5–6 replicate batches were transferred onto a chip. IJs were allowed to acclimate to the chip for 20–30 minutes. IJs were then assayed for waving or nictation behavior, as defined by the lifting of at least 30 % of the anterior of their body, during a 60 s period. IJs were counted as “nictating” if they were waving or nictating for a span of at least 5 s during the assay period.

### CO_2_ chemotaxis assay on micro-dirt agar chips

CO_2_ assays were as described above, except that they were conducted on 8 × 4 cm micro-dirt chips. IJs (*n* = 10–15) were allowed to acclimate to room temperature for 1 hour and then placed at the center of the chip and tested for their response to 10 % CO_2_. A modified scoring method was used in which the plate was divided into three equidistant regions along the diameter of the plate; IJs were counted as responding if they were found in the one-third of the chip under the CO_2_ flow.

### Statistical analysis

Statistical analysis was performed using GraphPad Prism 6.04. Standard statistical tests were used for all experiments, as described in the figure legends. For most figures, two-way ANOVAs were conducted, testing the effects of odorant and temperature/age. Multiple comparison post-tests were conducted if the interaction or temperature/age effect was significant. For all population assays, the value for sample size (*n*) refers to the number of trials performed for each condition. For Fig. 6b, because individual worms were monitored, responses were compiled in a contingency table and analyzed using Fisher’s exact test.

### Availability of data and materials

Mean, *n*, and standard error of the mean values for each assay are listed in Additional file 7: Dataset S1.

## Additional files

Additional file 1: Figure S1. Chemotaxis assay for infective juveniles (IJs). Odorants were placed on one side of the plate and controls were placed on the other side (black dots). IJs were placed in the center of the plate and allowed to migrate in the odorant gradient for 3 hours. A chemotaxis index was then calculated as indicated. The chemotaxis index ranges from +1 (most attractive) to –1 (most repulsive). Red bar = 1 cm. (PDF 289 kb) Additional file 2: Figure S2. Cultivation temperature, not assay temperature, modulates *Steinernema carpocapsae* olfactory behavior gradually across temperature ranges. A. *Ste. carpocapsae* IJs were cultivated at 15 °C, 20 °C, room temperature (22 ± 1 °C), or 25 °C and tested 21 days after host-emergence. Olfactory behaviors changed gradually and consistently within the temperature range; *n* = 6–20 trials for each condition. B. IJs were cultivated at 15 °C and assayed at day 28 at either room temperature or 15 °C. IJs exhibited slightly more robust responses when assayed at 15 °C, but the valence of the response to each odorant was the same regardless of the assay temperature. * *P* < 0.05, two-way ANOVA with Sidak’s post-test; *n* = 6 trials for each condition. C. 25 °C IJs that were temperature-swapped to 15 °C (right) or maintained at 25 °C (left) for 2 weeks were assayed at room temperature or the incubation temperature. A valence change was observable regardless of the assay temperature. Although the assay temperature did not affect valence, the temperature-swapped IJs showed a weaker response to 1-hexanol when assayed at room temperature compared to 15 °C. *** *P <* 0.001, two-way ANOVA with Sidak’s post-test; *n* = 6–10 trials for each condition. For all graphs, error bars represent standard error of the mean (SEM). Mean, n, and SEM values for each assay are listed in Additional file 7: Dataset S1. (PDF 415 kb) Additional file 3: Figure S3. Temperature-induced changes in sensory valence are consistent across CO_2_ and odorant concentrations in *Steinernema carpocapsae*. A. Responses of infective juveniles (IJs) to various CO_2_ concentrations. CO_2_ was attractive for IJs across concentrations regardless of age or temperature treatment, although 25 °C IJs showed an increased response to 0.5 % CO_2_ at day 14 compared to day 0. * *P <* 0.05 relative to day 0 IJs; two-way ANOVA with Dunnett’s post-test; *n* = 6–10 trials for each condition. B. Responses to different concentrations of 1-hexanol and 2-propanone were compared for day 0 IJs maintained at 25 °C, day 14 IJs that were temperature-swapped from 25 °C to 15 °C on day 0, and day 14 IJs maintained at 25 °C. Responses of consistent valence were observed across concentrations. Data for undiluted concentrations are from Fig. 3. Responses of 25 °C IJs to 2-propanone were significantly different between day 0 and day 14 at 10^–4^ (*P <* 0.05) and 10^–5^ (*P <* 0.01) concentrations in a two-way ANOVA with Sidak’s post-test, comparing 10^0^ through 10^–6^ dilutions between 25 °C day 0 and 25 °C day 14 IJs; n = 6–22 trials for each condition. C. IJs exhibit olfactory plasticity in response to odor blends. IJs were tested for their response to three different mixes of odorants. Mix 1 contained 10^–1^ dilutions of methyl acetate, 1-propanol, and 2-propanone; Mix 2 contained 10^–1^ dilutions of 1-hexanol, 6-methyl-5-hepten-2-one, and methyl salicylate; and Mix 3 contained 10^–1^ dilutions of 1-hexanol, 7-octenoic acid, and 2,2,4-trimethylpentane. *** *P* < 0.001 relative to day 0 IJs; two-way ANOVA with Dunnett’s post-test; *n* = 8 trials for each condition. For all graphs, error bars represent standard error of the mean (SEM). Mean, *n*, and SEM values for each assay are listed in Additional file 7: Dataset S1. (PDF 428 kb) Additional file 4: Figure S4. Olfactory plasticity occurs in individual infective juveniles (IJs), is not affected by cultivation density, and occurs in multiple strains of *Steinernema carpocapsae*. A. Temperature-induced changes in sensory valence occur in individual IJs. 25 °C IJs that were repelled by 2-propanone on day 0 were collected and cultured at either 15 °C or 25 °C for 2 weeks, and then re-tested on day 14 using a modified scoring method (left). The IJs that were temperature-swapped from 25 °C to 15 °C showed opposite olfactory preferences compared to those maintained at 25 °C. *** *P* < 0.001, unpaired *t*-test; *n* = 6 trials for each condition. Red bar = 1 cm. B. Cultivation density does not affect temperature-induced sensory valence changes; 25 °C day 0 *Ste. carpocapsae* IJs were collected and stored at 15 °C at low density (1 IJ/μL), medium density (6 IJ/μL), or high density (25 IJ/μL) and tested for their response to 2-propanone and 1-hexanol after 2 weeks of storage. No significant effects of cultivation density (*F*_2,62_ = 0.2586, *P =* 0.7730) or interaction (*F*_*2*,62_ = 1.912, *P* = 0.1565) were observed in a two-way ANOVA; n = 8–18 trials for each condition. C. Multiple strains of *Ste. carpocapsae* exhibit temperature-dependent olfactory plasticity. In addition to the standard All strain, the DD136 and Sal strains [101] also exhibited temperature-induced sensory valence changes. A comparison of day 0 IJs that were cultured at 25 °C, day 14 IJs that were temperature-swapped from 25 °C to 15 °C on day 0, and day 14 IJs that were cultured at 25 °C revealed both temperature- and age-dependent changes in olfactory responses. ** *P* < 0.01; *** *P* < 0.001 relative to 25 °C day 0 IJs, two-way ANOVA with Dunnett’s post-test; *n* = 6–16 trials for each condition. For all graphs, error bars represent standard error of the mean (SEM). Mean, *n*, and SEM values for each assay are listed in Additional file 7: Dataset S1. (PDF 529 kb) Additional file 5: Figure S5. Temperature-induced valence changes in olfactory responses exhibit long-term stability. *Steinernema carpocapsae* infective juveniles (IJs) were infected at room temperature and stored at 15 °C for up to 16 weeks. Their responses to a five-odor panel were recorded every 2 weeks after emergence from the host; *n* = 4–12 trials for each condition. Error bars represent standard error of the mean (SEM). Mean, *n*, and SEM values for each assay are listed in Additional file 7: Dataset S1. (PDF 341 kb) Additional file 6: Figure S6. Temperature and age affect olfactory behavior differently across entomopathogenic nematode species. *Steinernema carpocapsae* (A), *Heterorhabditis bacteriophora* (B), *Ste. scapterisci* (C), *Ste. riobrave* (D), *H. indica* (E), and *Ste. feltiae* (F) IJs were grown at 25 °C, and either temperature-swapped to 15 °C on day 0 or maintained at 25 °C. All six species show age- and/or temperature-dependent changes in olfactory responses. In (A), data for 1-hexanol is from Fig. 3. In (B), data is from Fig. 4. * *P* < 0.05; ** *P* < 0.01; *** *P* < 0.001 relative to 25 °C day 0 IJs, two-way ANOVA with Dunnett’s post-test; *n* = 6–18 trials for each condition. For all figures, error bars represent standard error of the mean (SEM). Mean, *n*, and SEM values for each assay are listed in Additional file 7: Dataset S1. (PDF 446 kb) Additional file 7: Dataset S1. Compilation of mean, *n*, and standard error of the mean (SEM) values for all assays presented in this study. (XLSX 57 kb)
