# Supplementary material for: Temperature-dependent changes in the host-seeking behaviors of parasitic nematodes
Source: BMC Biol. 2016 May 6;14:36. doi: 10.1186/s12915-016-0259-0 (PMC4858831; doi:10.1186/s12915-016-0259-0)
Supplement: Additional file 1: Figure S1. — Chemotaxis assay for infective juveniles (IJs). Odorants were placed on one side of the plate and controls were placed on the other side (black dots). IJs were placed in the center of the plate and allowed to migrate in the odorant gradient for 3 hours. A chemotaxis index was then calculated as indicated. The chemotaxis index ranges from +1 (most attractive) to –1 (most repulsive). Red bar = 1 cm. (PDF 289 kb) [file 12915_2016_259_MOESM1_ESM.pdf]

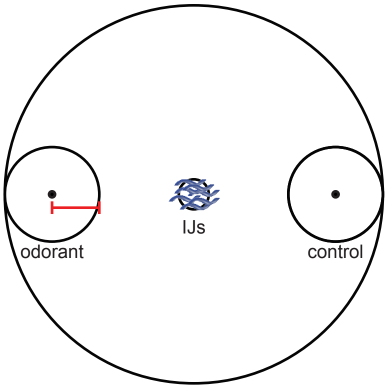


$$\text{chemotaxis index} = \frac{(\text{\# of IJs at odorant}) - (\text{\# of IJs at control})}{(\text{\# of IJs at odorant}) + (\text{\# of IJs at control})}$$
