# Supplementary figures and images for: Temperature-dependent changes in the host-seeking behaviors of parasitic nematodes
Source: BMC Biol. 2016 May 6;14:36. doi: 10.1186/s12915-016-0259-0 (PMC4858831; doi:10.1186/s12915-016-0259-0)

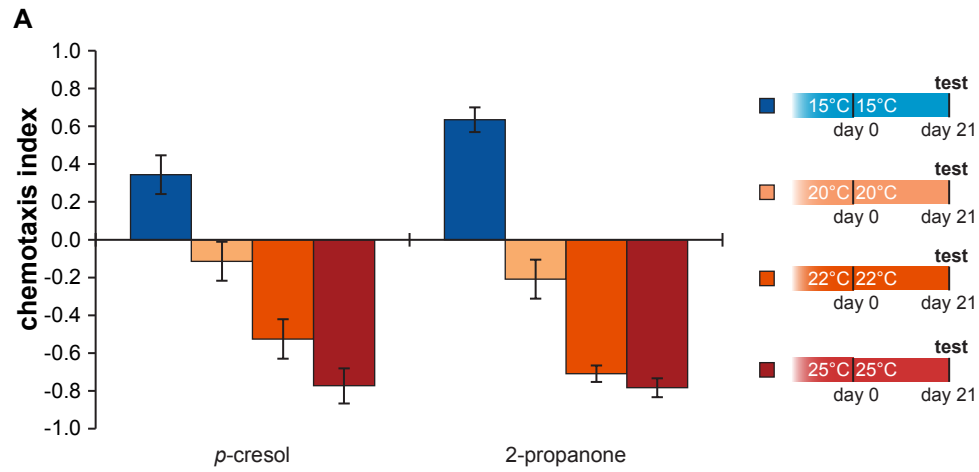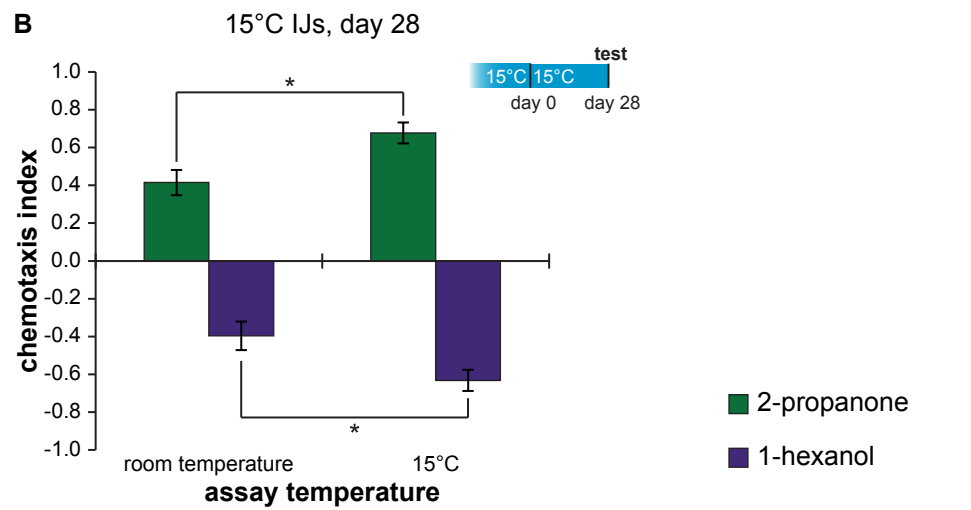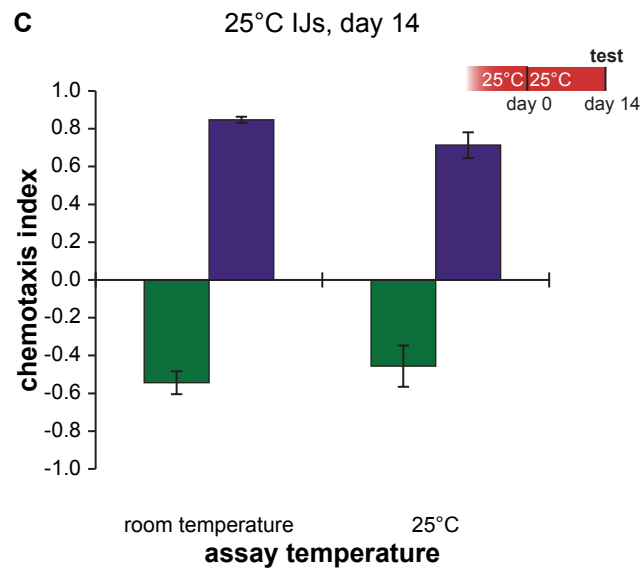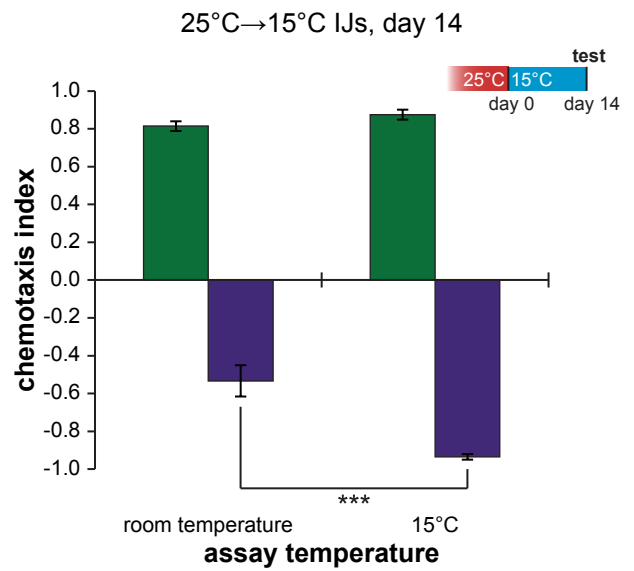

Supplement: Additional file 2: Figure S2. — Cultivation temperature, not assay temperature, modulates Steinernema carpocapsae olfactory behavior gradually across temperature ranges. A. Ste. carpocapsae IJs were cultivated at 15 °C, 20 °C, room temperature (22 ± 1 °C), or 25 °C and tested 21 days after host-emergence. Olfactory behaviors changed gradually and consistently within the temperature range; n = 6–20 trials for each condition. B. IJs were cultivated at 15 °C and assayed at day 28 at either room temperature or 15 °C. IJs exhibited slightly more robust responses when assayed at 15 °C, but the valence of the response to each odorant was the same regardless of the assay temperature. * P < 0.05, two-way ANOVA with Sidak’s post-test; n = 6 trials for each condition. C. 25 °C IJs that were temperature-swapped to 15 °C (right) or maintained at 25 °C (left) for 2 weeks were assayed at room temperature or the incubation temperature. A valence change was observable regardless of the assay temperature. Although the assay temperature did not affect valence, the temperature-swapped IJs showed a weaker response to 1-hexanol when assayed at room temperature compared to 15 °C. *** P < 0.001, two-way ANOVA with Sidak’s post-test; n = 6–10 trials for each condition. For all graphs, error bars represent standard error of the mean (SEM). Mean, n, and SEM values for each assay are listed in Additional file 7: Dataset S1. (PDF 415 kb) [file 12915_2016_259_MOESM2_ESM.pdf]

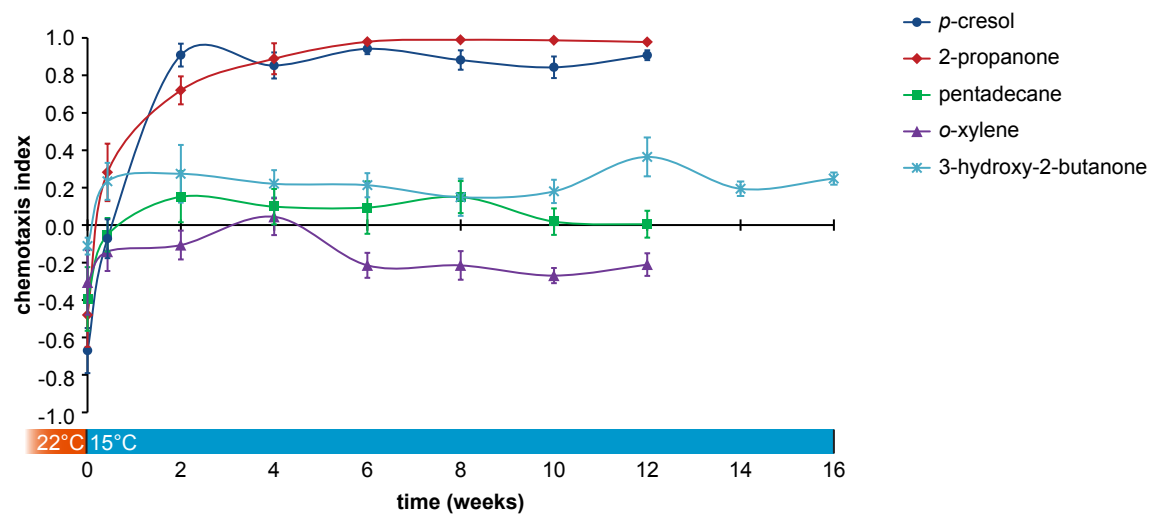

Supplement: Additional file 5: Figure S5. — Temperature-induced valence changes in olfactory responses exhibit long-term stability. Steinernema carpocapsae infective juveniles (IJs) were infected at room temperature and stored at 15 °C for up to 16 weeks. Their responses to a five-odor panel were recorded every 2 weeks after emergence from the host; n = 4–12 trials for each condition. Error bars represent standard error of the mean (SEM). Mean, n, and SEM values for each assay are listed in Additional file 7: Dataset S1. (PDF 341 kb) [file 12915_2016_259_MOESM5_ESM.pdf]

**A** *Ste. carpocapsae*

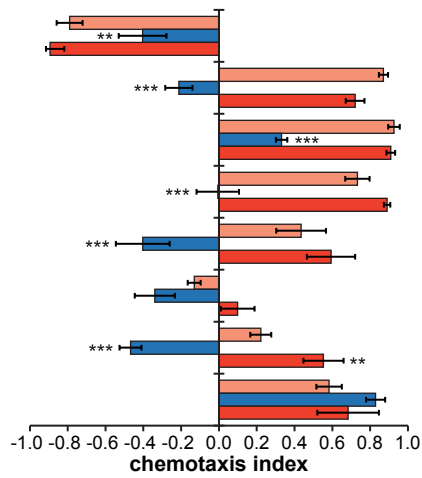

**B** *H. bacteriophora*

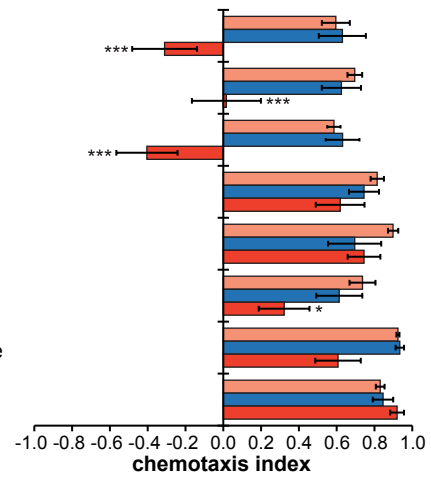

**C** *Ste. scapterisci*

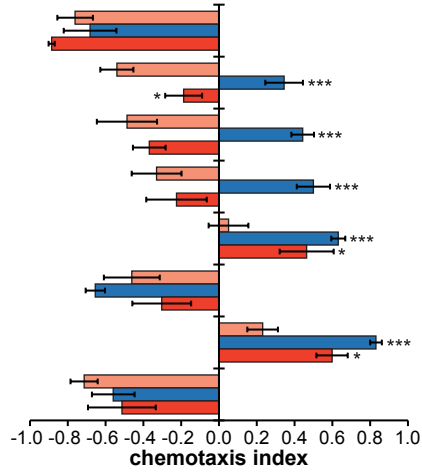

**D** *Ste. riobrave*

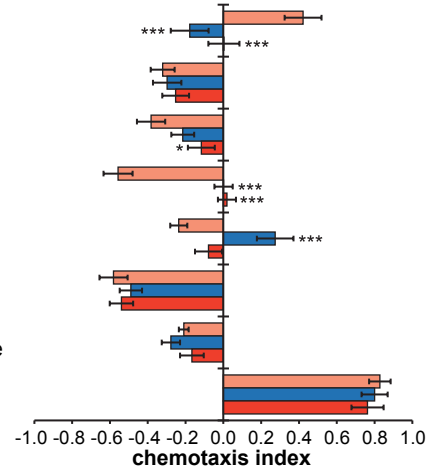

**E** *H. indica*

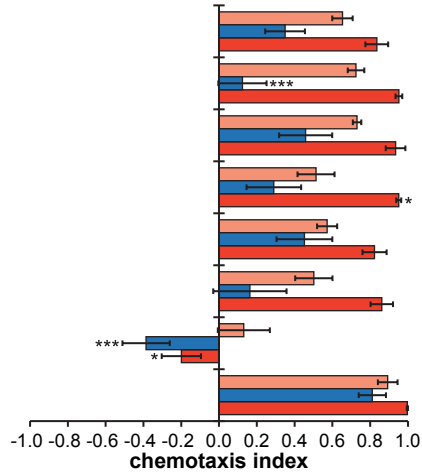

**F** *Ste. feltiae*

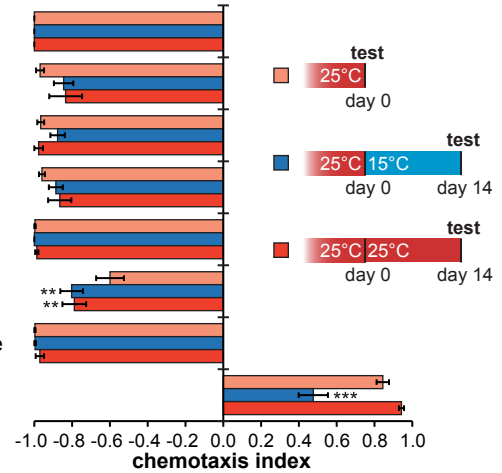

Supplement: Additional file 6: Figure S6. — Temperature and age affect olfactory behavior differently across entomopathogenic nematode species. Steinernema carpocapsae (A), Heterorhabditis bacteriophora (B), Ste. scapterisci (C), Ste. riobrave (D), H. indica (E), and Ste. feltiae (F) IJs were grown at 25 °C, and either temperature-swapped to 15 °C on day 0 or maintained at 25 °C. All six species show age- and/or temperature-dependent changes in olfactory responses. In (A), data for 1-hexanol is from Fig. 3. In (B), data is from Fig. 4. * P < 0.05; ** P < 0.01; *** P < 0.001 relative to 25 °C day 0 IJs, two-way ANOVA with Dunnett’s post-test; n = 6–18 trials for each condition. For all figures, error bars represent standard error of the mean (SEM). Mean, n, and SEM values for each assay are listed in Additional file 7: Dataset S1. (PDF 446 kb) [file 12915_2016_259_MOESM6_ESM.pdf]
