# Supplementary material for: Temperature-dependent changes in the host-seeking behaviors of parasitic nematodes
Source: BMC Biol. 2016 May 6;14:36. doi: 10.1186/s12915-016-0259-0 (PMC4858831; doi:10.1186/s12915-016-0259-0)
Supplement: Additional file 3: Figure S3. — Temperature-induced changes in sensory valence are consistent across CO2 and odorant concentrations in Steinernema carpocapsae. A. Responses of infective juveniles (IJs) to various CO2 concentrations. CO2 was attractive for IJs across concentrations regardless of age or temperature treatment, although 25 °C IJs showed an increased response to 0.5 % CO2 at day 14 compared to day 0. * P < 0.05 relative to day 0 IJs; two-way ANOVA with Dunnett’s post-test; n = 6–10 trials for each condition. B. Responses to different concentrations of 1-hexanol and 2-propanone were compared for day 0 IJs maintained at 25 °C, day 14 IJs that were temperature-swapped from 25 °C to 15 °C on day 0, and day 14 IJs maintained at 25 °C. Responses of consistent valence were observed across concentrations. Data for undiluted concentrations are from Fig. 3. Responses of 25 °C IJs to 2-propanone were significantly different between day 0 and day 14 at 10–4 (P < 0.05) and 10–5 (P < 0.01) concentrations in a two-way ANOVA with Sidak’s post-test, comparing 100 through 10–6 dilutions between 25 °C day 0 and 25 °C day 14 IJs; n = 6–22 trials for each condition. C. IJs exhibit olfactory plasticity in response to odor blends. IJs were tested for their response to three different mixes of odorants. Mix 1 contained 10–1 dilutions of methyl acetate, 1-propanol, and 2-propanone; Mix 2 contained 10–1 dilutions of 1-hexanol, 6-methyl-5-hepten-2-one, and methyl salicylate; and Mix 3 contained 10–1 dilutions of 1-hexanol, 7-octenoic acid, and 2,2,4-trimethylpentane. *** P < 0.001 relative to day 0 IJs; two-way ANOVA with Dunnett’s post-test; n = 8 trials for each condition. For all graphs, error bars represent standard error of the mean (SEM). Mean, n, and SEM values for each assay are listed in Additional file 7: Dataset S1. (PDF 428 kb) [file 12915_2016_259_MOESM3_ESM.pdf]

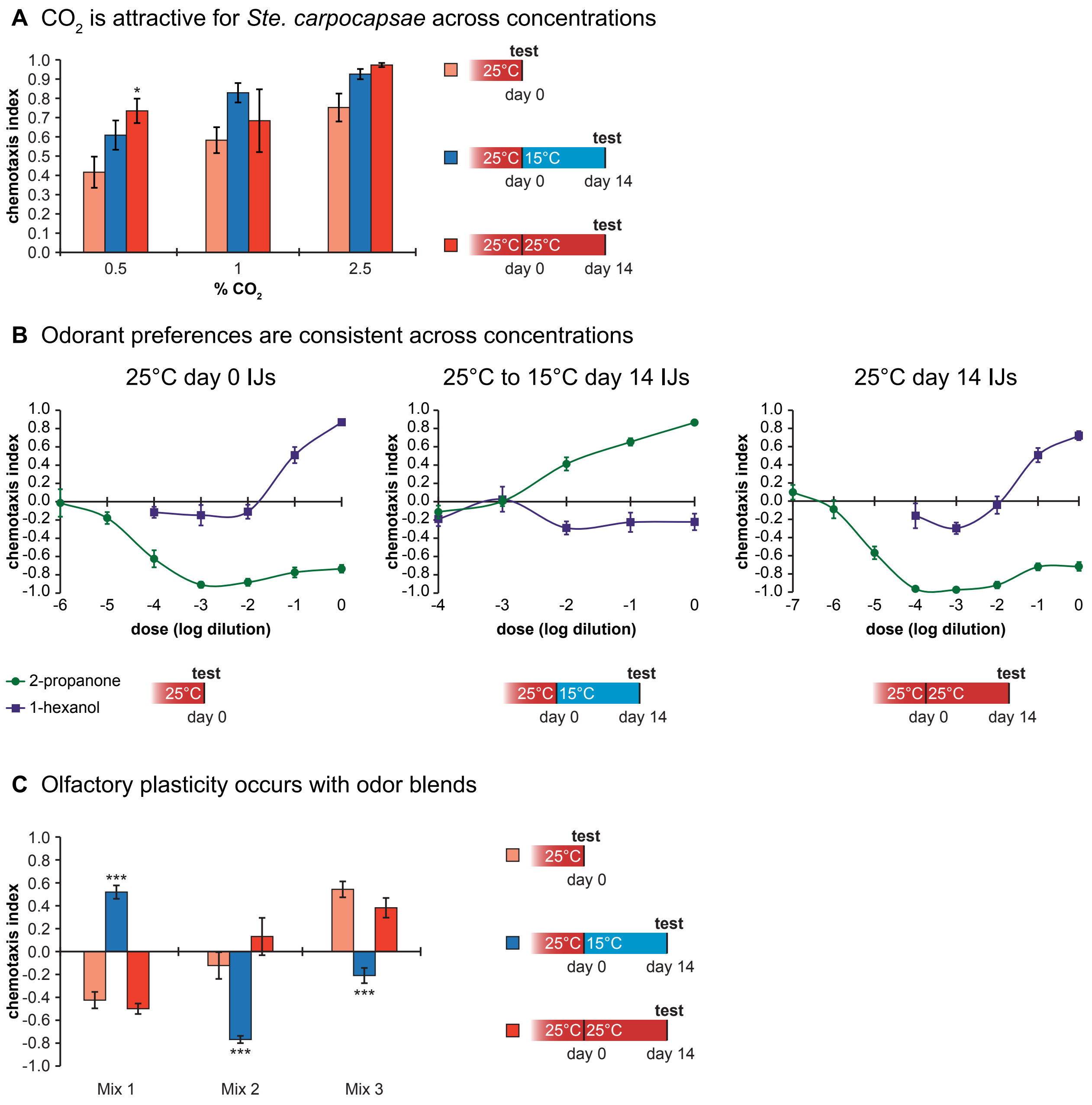
A CO2 is attractive for Ste. carpocapsae across concentrations
chemotaxis index
1.0
0.9
0.8
0.7
0.6
0.5
0.4
0.3
0.2
0.1
0.0
*
0.5
1
2.5
% CO2
test
25°C
day 0
test
25°C 15°C
day 0 day 14
test
25°C 25°C
day 0 day 14
B Odorant preferences are consistent across concentrations
25°C day 0 IJs
25°C to 15°C day 14 IJs
25°C day 14 IJs
chemotaxis index
1.0
0.8
0.6
0.4
0.2
0.0
-0.2
-0.4
-0.6
-0.8
-1.0
-7
-6
-5
-4
-3
-2
-1
0
dose (log dilution)
2-propanone
1-hexanol
test
25°C
day 0
test
25°C 15°C
day 0 day 14
test
25°C 25°C
day 0 day 14
C Olfactory plasticity occurs with odor blends
chemotaxis index
1.0
0.8
0.6
0.4
0.2
0.0
-0.2
-0.4
-0.6
-0.8
-1.0
***
***
***
Mix 1
Mix 2
Mix 3
test
25°C
day 0
test
25°C 15°C
day 0 day 14
test
25°C 25°C
day 0 day 14
