# Supplementary material for: Temperature-dependent changes in the host-seeking behaviors of parasitic nematodes
Source: BMC Biol. 2016 May 6;14:36. doi: 10.1186/s12915-016-0259-0 (PMC4858831; doi:10.1186/s12915-016-0259-0)

A Olfactory plasticity occurs in individual IJs

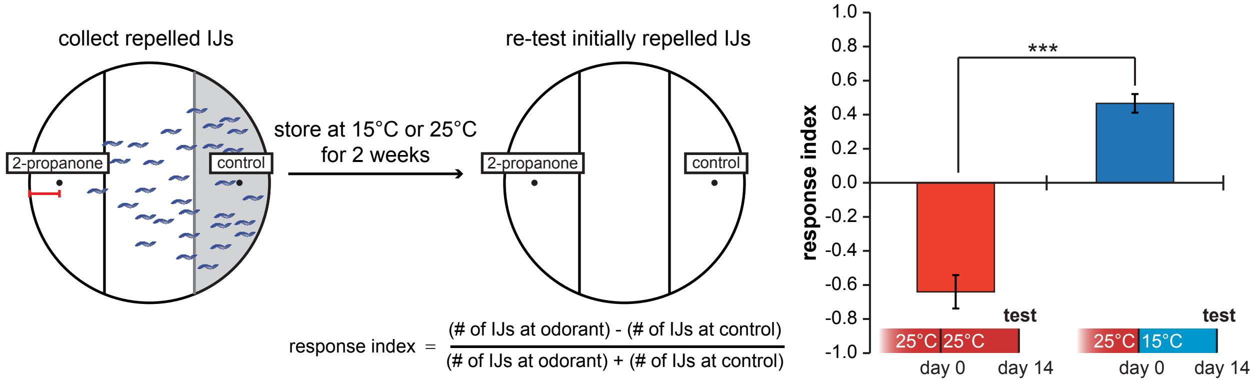

B Cultivation density does not affect olfactory responses

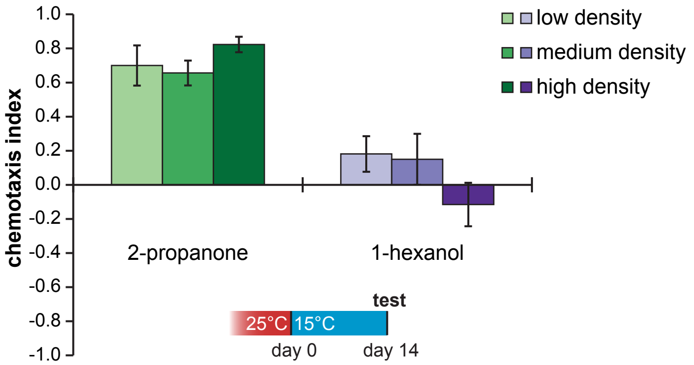

C Multiple strains of *Ste. carpocapsae* exhibit olfactory plasticity

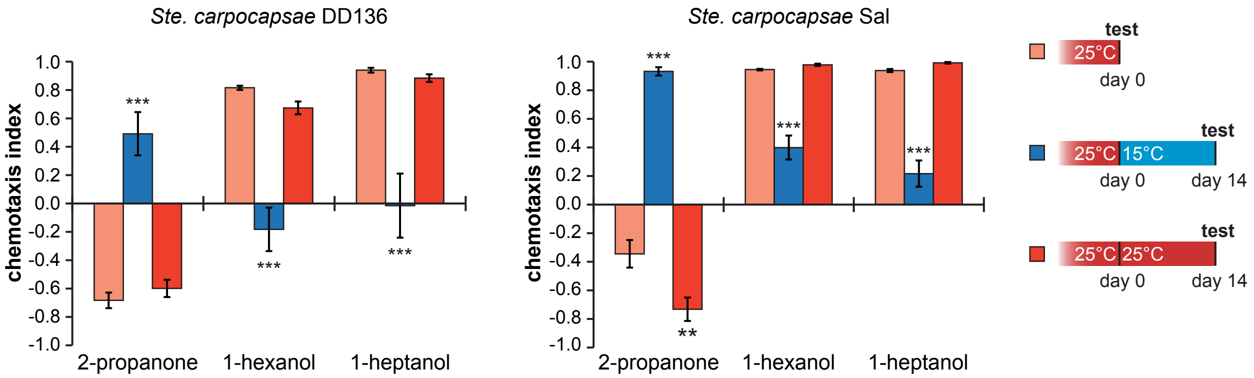

Supplement: Additional file 4: Figure S4. — Olfactory plasticity occurs in individual infective juveniles (IJs), is not affected by cultivation density, and occurs in multiple strains of Steinernema carpocapsae. A. Temperature-induced changes in sensory valence occur in individual IJs. 25 °C IJs that were repelled by 2-propanone on day 0 were collected and cultured at either 15 °C or 25 °C for 2 weeks, and then re-tested on day 14 using a modified scoring method (left). The IJs that were temperature-swapped from 25 °C to 15 °C showed opposite olfactory preferences compared to those maintained at 25 °C. *** P < 0.001, unpaired t-test; n = 6 trials for each condition. Red bar = 1 cm. B. Cultivation density does not affect temperature-induced sensory valence changes; 25 °C day 0 Ste. carpocapsae IJs were collected and stored at 15 °C at low density (1 IJ/μL), medium density (6 IJ/μL), or high density (25 IJ/μL) and tested for their response to 2-propanone and 1-hexanol after 2 weeks of storage. No significant effects of cultivation density (F 2,62 = 0.2586, P = 0.7730) or interaction (F 2,62 = 1.912, P = 0.1565) were observed in a two-way ANOVA; n = 8–18 trials for each condition. C. Multiple strains of Ste. carpocapsae exhibit temperature-dependent olfactory plasticity. In addition to the standard All strain, the DD136 and Sal strains [101] also exhibited temperature-induced sensory valence changes. A comparison of day 0 IJs that were cultured at 25 °C, day 14 IJs that were temperature-swapped from 25 °C to 15 °C on day 0, and day 14 IJs that were cultured at 25 °C revealed both temperature- and age-dependent changes in olfactory responses. ** P < 0.01; *** P < 0.001 relative to 25 °C day 0 IJs, two-way ANOVA with Dunnett’s post-test; n = 6–16 trials for each condition. For all graphs, error bars represent standard error of the mean (SEM). Mean, n, and SEM values for each assay are listed in Additional file 7: Dataset S1. (PDF 529 kb) [file 12915_2016_259_MOESM4_ESM.pdf]
